# Excess success for three related papers on racial bias

**DOI:** 10.3389/fpsyg.2015.00512

**Published:** 2015-05-01

**Authors:** Gregory Francis

**Affiliations:** ^1^Department of Psychological Sciences, Purdue University, West Lafayette, INUSA; ^2^Brain Mind Institute, École Polytechnique Fédérale de Lausanne, LausanneSwitzerland

**Keywords:** errors, excess success, racial bias, publication bias, statistics, theory

## Abstract

Three related articles reported that racial bias altered perceptual experience and influenced decision-making. These findings have been applied to training programs for law enforcement, and elsewhere, to mitigate racial bias. However, a statistical analysis of each of the three articles finds that the reported experimental results should be rare, even if the theoretical ideas were correct. The analysis estimates that the probability of the reported experimental success for the articles is 0.003, 0.048, and 0.070, respectively. These low probabilities suggest that similar future work is unlikely to produce as successful outcomes and indicates that readers should be skeptical about the validity of the reported findings and their theoretical implications. The reported findings should not be used to guide policies related to racial bias, and new experimental work is needed to judge the merit of the theoretical ideas.

## Introduction

Over the past decade, Eberhardt and colleagues published sets of findings that concluded racial bias alters perceptual percepts and influences behavior and decision-making in a variety of important ways (such as sentences for criminal violations). These findings address a very important topic, and they have been hailed as having important implications for guiding policy and training in police departments and elsewhere (Laszlo and Fridell, 2012; MacArthur Foundation, 2014; Dreifus, 2015; Noë, 2015).

The statistical analyses described below indicate that the findings in several of Eberhardt’s articles seem too successful relative to what could be expected for the reported size of the effects and the design of the experiments (Ioannidis and Trikalinos, 2007; Francis, 2012, 2013a, 2014a; Schimmack, 2012), which means scientists should be skeptical about the reported theoretical conclusions and that it may be premature to use these conclusions to inform policy. Before turning to the analyses of the articles, it may be valuable to describe the general method of the analysis, which is called the test for excess success (TES).

The theoretical claims about racial bias in the original articles were based on standard hypothesis tests. Hypothesis tests are designed to control the probability of incorrectly concluding the presence of an effect (such as population means being different) when it does not exist (the population means are equal). Such control is necessary because even when population means are truly equal the means of samples randomly drawn from those populations are almost never exactly equal. To claim statistical significance, the difference of sample statistics must be sufficiently large to limit the probability of mistakenly concluding that an effect exists when in reality it does not (this mistake is called a Type I error). Typically, the criterion for judging significance is set so that the Type I error rate across repeated experiments is 0.05.

Similarly, even when population means are truly different, some randomly chosen samples will not produce a significant difference. To not conclude a significant effect when it really does exist is to make a Type II error. The complement of the Type II error rate is sometimes called power: the probability that randomly selected samples in an experiment will reject the null hypothesis when the effect is real. Scientists want experiments with high power, but unlike the control of Type I error, which is established by setting the criterion for judging significance, power depends on the size of the true effect and on the sample sizes. Since, the true effect size is generally unknown (else the experiment would not be run), it is difficult to precisely control Type II error, so scientists have to do the best they can by using theoretical ideas, past experience, or intuition (Vankov et al., 2014) to balance the increased experimental power that comes with a larger sample size against the costs of time and money that are spent when acquiring a large sample.

At best hypothesis testing can control, but not eliminate, the probability of making decision errors. Thus, the very nature of hypothesis testing is such that scientists are bound to sometimes make statistical errors in their decisions. For any given experiment, a scientist never knows if they have made an error or a correct decision, but over multiple experiments errors become ever more likely. A troubling aspect for some studies in experimental psychology is that, relative to the corresponding theoretical claims, such errors largely seem to be missing from the published record.

Using the published experimental results, it is possible to estimate the probability that an experiment like the one reported would produce a “successful” outcome. Depending on the theoretical claims, success may be a significant result, it may be a non-significant result, or it may be a pattern of significant and non-significant results. These probability values can be used to compute *P_TES_*, the estimated probability that a replication of all of the experiments in the set (using the same sample sizes) would produce outcomes at least as successful as what was originally reported. Ioannidis and Trikalinos (2007) considered a special case where success was always to produce a significant result and all experiments measured the same effect. The present analysis considers a broader combination of outcomes and heterogeneous effects that are related to a given set of theoretical conclusions (Francis, 2013a).

If the *P_TES_* value is low (a common criterion is 0.1) then scientists should be skeptical about the originally reported outcomes, because even if the effects are real it is surprising that the reported experiments would be so successful. Such experiment sets have *excess success*. The 0.1 criterion is conservative in the sense that most scientists would be unsatisfied if the set of experimental findings used to support their theoretical conclusions replicated only 10% of the time. Indeed, since replication is considered a gold standard of experimental validation, many scientists would be concerned if their findings replicated only 50% of the time.

A set of experimental findings can have an excess of success for a variety of reasons (Simmons et al., 2011). Although there are minor variations, the reasons come in four main classes.

(1) *Publication bias.* Authors may run unsuccessful experiments but not publish them. In such a case the published experimental results give a false impression of the magnitude and reliability of the effects. They also give a false impression about the validity of the theoretical ideas. Not reporting unsuccessful outcomes often produces an experiment set having an excess of successful outcomes.
(2) *Improper sampling:* To control error rates, standard hypothesis testing requires drawing random samples with a fixed size. One improper sampling approach is to not use a fixed size, but to sample and test until a desired outcome is produced. For example, a scientist might gather data from 15 subjects in each group and run a hypothesis test that produces *p* = 0.07. This result does not satisfy the typical criterion for statistical significance, so the scientist adds an additional five subjects to each group and runs the test again. If the new test produces *p* < 0.05, a significant outcome is reported; but if the new test still produces a non-significant outcome, the scientist may add more subjects or stop the experiment. This sampling approach dramatically inflates the Type I error rate (the problem is made worse if the unsuccessful outcomes are not published), and it also generates an excess of successful outcomes across a set of experiments.
(3) *Improper analysis:* Oftentimes data sets can be analyzed in a variety of ways. For example, a scientist might first analyze data with an analysis of variance (ANOVA), which makes certain assumptions about the properties of the data populations. Should an unsuccessful result be found (*p* > 0.05), the scientist might transform the data and try again, run a different test that takes into account other variables, remove some data points for being outliers, or split the data in various ways to allow for other types of tests. Trying out different types of analysis methods increases the probability of making a Type I error, and it also generates an excess of successful outcomes for a set of experiments.
(4) *Theory over fitting:* As noted above, experiments with real effects will sometimes not show those effects and experiments with no effect will sometimes show evidence for an effect. This uncertainty means that if a theory is derived to match patterns of significant and non-significant outcomes from experimental data that only modestly satisfies the significance criterion, then the theory is very likely to include some characteristics that are due to noise introduced by random sampling. Such a theory creation process labels both significant and non-significant outcomes as part of the theory, and it generates excess success across a set of experiments.

Regardless of the reason for its appearance, excess success across a set of experiments suggests some kind of problem in the experiment set as it relates to the theoretical conclusions. As such, its presence means scientists should be skeptical about the data, the analyses, or the theoretical ideas. Of course, excess success can occur by chance (an unlucky scientist will sometimes randomly choose samples that happen to produce more successful outcomes than would be expected), but scientists should still be skeptical about such experiment sets because they appear to be too successful and because such unlucky outcomes tend to not represent reality.

Francis (2013a) provides further details and properties of the TES. Accompanying commentaries (Gelman, 2013; Ioannidis, 2013; Johnson, 2013; Morey, 2013; Simonsohn, 2013; Vandekerckhove et al., 2013) and a response to the commentaries (Francis, 2013b) further discuss some possible limitations and characteristics of a TES analysis.

Many previous studies have found evidence that publication bias is common across psychology and other fields (e.g., Sterling, 1959; Sterling et al., 1995; Bakker et al., 2012; Fraley and Vazire, 2014; Francis, 2014a,b; Kühberger et al., 2014). Given the existence of publication bias across the field, and newly developed methods of adjusting for it in some situations (e.g., van Assen et al., 2014), it is appropriate to consider whether it is necessary to investigate publication bias for a specific subset of experimental studies. Three arguments suggest that the answer is “yes.”

First, some forms of bias do not necessarily undermine the relationship between published data and an author’s theoretical conclusions. In particular, it is possible for a biased field to be made up of experimental subsets that are unbiased relative to the theoretical conclusions associated with those subsets. For example, suppose there are two independent projects that investigate how people use keyboards and how people see visual afterimages. The findings and theoretical conclusions from the afterimage studies are not undermined if the keyboard studies are suppressed, even though such suppression introduces publication bias when considering both sets. An experimental scientist’s role is not to produce uniformly unbiased experimental outcomes across all topics, but to provide convincing experimental findings that support (or refute) theoretical ideas. Thus, even when a field is known to have some bias, it remains necessary to explore subsets of that field to check on bias relative to the corresponding theoretical claims.

Second, the appearance of bias in an experiment set undermines the corresponding theoretical conclusions, so scientists should be cautious about applying those conclusions outside the laboratory. If true, the theoretical ideas proposed by Eberhardt and colleagues about racial bias have many applications to important situations in society; but attempts to mitigate racial bias or to reduce its presence will likely fail if they are based on conclusions derived from unconvincing evidence. When sets of experimental findings are promoted as being especially important, it is appropriate and necessary for scientists to re-evaluate them. The three studies analyzed below have been promoted in this way (MacArthur Foundation, 2014).

Third, although techniques have been developed to perform a fixed effect meta-analysis under conditions of publication bias (van Assen et al., 2014) and for identifying whether biased experiment sets contain some evidential value (Simonsohn et al., 2014), these methods do not apply to the work of Eberhardt et al. (2004) because, as described below, the experiments measured widely differing effects and because a successful outcome within an experiment often involves multiple hypothesis tests. Moreover, in many situations the relevant scientific question is not whether there is some evidential value in the reported experiments, but whether the presented theoretical conclusions are supported by the presented experimental results. Even if a set of experiments has some evidential value it still might not support the theoretical claims.

It is important to emphasize that the TES analyses in this paper investigate the relationship between experimental findings and theoretical conclusions. The analyses do not demonstrate that the original authors acted in a dishonest manner or committed scientific misconduct. Until proven otherwise, the appearance of excess success should be taken to mean that the original authors appear to have made decisions in their analyses or reporting that could have inflated Type I error rates or effect size estimates. Such mistakes are hardly rare in the field of psychology, and they need to be identified and recognized, when possible.

## Materials and Methods

Article selection was motived by the MacArthur Foundation referring to Eberhardt’s studies on racial bias as motivation for awarding her a “genius” fellowship (MacArthur Foundation, 2014). On 17 September 2014, the web site (http://web.stanford.edu/group/mcslab/cgi-bin/wordpress/publications/) for the “Mind, Culture, and Society Lab at Stanford” listed Eberhardt as being an author or co-author of six articles from a list of “Selected Recent Publications.” The excess success analysis requires multiple studies (four is a reasonable minimum number), and three of the articles contained four or more studies. Each of these three articles is analyzed below in chronological order.

## Results

All of the analysis programs are provided as supplemental material.

### Eberhardt et al. (2004) “Seeing Black: Race, Crime, and Visual Processing”

This article reported five studies purporting to show that priming participants with Black faces or with concepts related to Black Americans changed properties of visual perception and attention to be more sensitive to stereotypes of Black Americans. **Table 1** summarizes the statistical measures for each study, the key hypotheses that were tested in the article, and the estimated probability of success for those hypothesis tests.

**Table 1:** Statistical properties, hypotheses, and estimated probability of success for the tests in the five studies from Eberhardt et al. (2004).

	Statistics	Supporting hypotheses	Probability of success
Study 1	*n*_1_ = 13, *n*_2_ = 12, *n*_3_ = 14 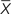 _*1A*_ = 26.9, 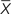 _*1B*_ = 24.1, *s*_*1A*_ = 4.51, *s*_*1B*_ = 4.76 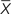 _*2A*_ = 23.0, 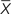 _*2B*_ = 23.2, *s*_*1A*_ = 4.57, *s*_*1B*_ = 4.57 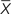 _*3A*_ = 18.3, 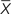 _*3B*_ = 22.7, *s*_*1A*_ = 4.81, *s*_*1B*_ = 4.68 *r*_*1AB*_ = 0.582 *r*_*2AB*_ = 0.302	μ_3A_ ≠ μ_1A_ μ_3A_ ≠ μ_2A_ μ_1A_ ≠ μ_2A_ μ_3B_ = μ_2B_ μ_1B_ = μ_2B_ μ_3A_ ≠ μ_3B_ μ_1A_ ≠ μ_1B_ Joint	0.996 0.680 0.541 0.944 0.926 0.780 0.582 **0.163**
Study 2	*n*_*1A*_ = 13, *n*_*2A*_ = 12, *n*_*1B*_ = 13, *n*_*2B*_ = 12 *d*_*A*,(1-2)_ = 1.09, *d*_*B*,(1-2)_ = 0.779	μ_1A_ ≠ μ_2A_ μ_1B_ ≠ μ_2B_* Joint	0.777 0.482 **0.380**
Study 3	*n*_*1A*_ = 17, *n*_*2A*_ = 17, *n*_*1B*_ = 17, *n*_*2B*_ = 18 *d*_*A*,(1-2)_ = 0.746	μ_1A_ ≠ μ_2A_	**0.575**
Study 4	*n*_*1A*_ = 14, *n*_*2A*_ = 14, *n*_*1B*_ = 14, *n*_*2B*_ = 15 *d*_*A*,(1-2)_ = 0.729, *d*_*B*,(1-2)_ = 1.282	μ_1A_ ≠ μ_2A_† μ_1B_ ≠ μ_2B_ Joint	0.484 0.924 **0.450**
Study 5	*n*_*1A*_ = 18, *n*_*2A*_ = 20, *n*_*1B*_ = 20, *n*_*2B*_ = 20 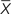 _1A_ = 10.83, 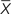 _*1B*_ = 10.50 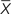 _*2A*_ = 12.95, 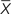 _*2B*_ = 8.80 *s* = 3.02	μ_1_ ≠ μ_2_ Interaction μ_1A_ ≠ μ_1B_ μ_2A_ = μ_2B_ Joint	0.723 0.563 0.569 0.796 **0.212**
*P_TES_*			0.003

*Eberhardt et al. (2004) concluded statistical significance for *p* < 0.055. †Eberhardt et al. (2004) concluded statistical significance for *p* < 0.053. *P_*TES*_* refers to the estimated probability of all experiments like these producing successful outcomes. A bold probability in the final column indicates the joint success probability for the corresponding study.

Study 1 found that priming participants to Black male faces (rather than White faces or no prime) increased sensitivity to crime-related objects (rather than crime-irrelevant objects) in a degraded image. This conclusion was based on seven hypothesis tests: (1) a significant difference between the Black and White primes for crime-related objects, (2) a significant difference between the Black and no prime conditions for crime-related objects, (3) a significant difference (in the opposite direction) between the White and no prime conditions for crime-related objects, (4) a predicted non-significant difference between the Black and no prime conditions for crime-irrelevant objects, (5) a predicted non-significant difference between the White and no prime conditions for crime-irrelevant objects, (6) a significant difference between crime-related and crime-irrelevant objects for participants primed by Black faces, and (7) a significant difference (in the opposite direction) between crime-related and crime-irrelevant objects for participants primed by White faces. The study also required a significant interaction of prime and relevance, but the reported statistics in Eberhardt et al. (2004) did not provide enough information to estimate the success probability for that test (a necessary correlation for one of the within-subject measures is not reported and cannot be derived from other statistics).

All of these tests produced results that appeared to support the theoretical ideas described in Eberhardt et al. (2004). However, with so many outcomes that must be satisfied with a single data set, such full success should be rare even if the effects are real and as estimated by the experimental data. To estimate the probability of such a level of success, a software program (R Core Team, 2014) was used to generate 100,000 simulated experiments with the sample sizes, means, standard deviations, and correlations (for within-subject aspects of the experiment) that were described in (or derived from) Eberhardt et al. (2004). **Table 1** shows that the success probability for any given hypothesis test varies between 0.541 and 0.996, but the joint probability that all of the tests would be successful in a single experiment is only 0.163 (this value is larger than the product of the success probabilities of individual tests because the tests are not independent).

Study 2 reported that participants primed by a crime were faster to detect a small dot on a Black face than on a White face. The study used a two (prime: crime or none) by two (dot position: Black or White face) between-subjects design. The theoretical ideas were supported by two significance tests: (1) a significant difference between primes for Black faces and (2) a significant difference (in the opposite direction) between primes for White faces. The second hypothesis test reported *p* = 0.05, but a recalculation for the given *F*-value indicates that *p* = 0.054, which is above the standard 0.05 criterion. Such *p*-value misreporting is not uncommon (Bakker and Wicherts, 2011; Wicherts et al., 2011). Regardless, researchers sometimes use an alternative significance criterion; and since Eberhardt et al. (2004) claimed success for this hypothesis test, their criterion was apparently something like 0.055 (a much larger criterion would generally not be accepted by reviewers). Identification of this criterion is necessary to estimate the probability of a successful outcome. Eberhardt et al. (2004) reported mean latencies but the analysis was based on an inverse-transform of the latencies (to remove apparent skewness in the data). As a result, the means of different groups cannot be estimated from the data in Eberhardt et al. (2004). However, the reported test statistics do allow computation of standardized effect sizes, which can be used to generate equivalent hypothesis tests. Eberhardt et al. (2004) also required (and found) a significant interaction, but the standardized effect sizes by themselves are insufficient to include that test among the reported successes. The standardized effect sizes are shown in **Table 1**.

Study 3 had a design similar to Study 2, but used a basketball prime rather than crime. Here, two hypothesis tests were deemed necessary for success: (1) a significant interaction and (2) a significant difference between primes when searching on a Black face. For reporting details similar to those in Study 2, it is not possible to estimate success probabilities for both of these tests (they are not independent), but it is possible to estimate the success probability of either one with the standardized effect size; and **Table 1** reports the probability for test (2). Eberhardt et al. (2004) did note that, contrary to Study 2, the effect of primes for White faces was not significantly different. This could have been interpreted as a non-success, but instead Eberhardt et al. (2004) chose to interpret the outcome as being due to differences in the nature of the priming method. At any rate, they did not treat the non-significant outcome as evidence against their theoretical ideas.

Study 4 was similar to Study 2 but used police officers as participants. The same limitations on the analysis as for Studies 2 and 3 also apply to Study 4. Although a total of five successful hypothesis tests were presented to support the theoretical ideas in Eberhardt et al. (2004), success probabilities can only be estimated for two of the tests: (1) a significant difference between primes for Black faces and (2) a significant difference (in the opposite direction) between primes for White faces. The first hypothesis was judged successful with a reported *p* < 0.05, but for the given *F*(1,53) = 3.95 the *p*-value is actually 0.052. Thus, for this test, success was apparently concluded for *p*-values less than 0.053, which is what was used for the success probability calculations.

Study 5 explored effects of race (Black or White) and racial stereotypicality (high or low) on judgments of criminality from facial observations. The theoretical ideas were said to be supported by four hypothesis tests: (1) a main effect of Black faces versus White faces, (2) a significant interaction between race and stereotypicality, (3) a significant difference between high and low stereotypicality for Black faces, and (4) a non-significant effect of stereotypicality for White faces. The text reported other tests, but they do not seem necessary for the theoretical ideas. The means and standard deviations were estimated from Figure 7 in Eberhardt et al. (2004). Recomputing the statistical tests revealed that none of the reported *F*-values in the text agreed with the reported means and standard deviations, although the patterns of significant and non-significant outcomes were identical. To reconcile this discrepancy, the means were estimated from Figure 7 and the standard deviation from the *F*-values in the text. The analysis by Eberhardt et al. (2004) was somewhat non-standard as data was gathered from a sample of 166 police officers but the sample groups used for the hypothesis tests were the faces. The estimated success probabilities assume that this non-standard analysis is valid and appropriate for the theoretical ideas. Although every hypothesis test has a success probability above one half, the probability of a sample satisfying all of these tests simultaneously is only 0.212.

If the effects are real and of similar magnitude to what is reported in the sample data, the experiments were run properly and analyzed properly, and all relevant studies were fully reported, then across all five studies, the probability of the level of success reported by Eberhardt et al. (2004) is the product of the joint probabilities for each study. This value is 0.003, meaning that the degree of success reported by Eberhardt et al. (2004) should be extremely rare, and future studies of the same phenomena should not show nearly the degree of success that is reported in the original study.

Indeed, the reported findings should be so rare that it is doubtful that the findings in Eberhardt et al. (2004) are representative of reality. If unsuccessful experimental outcomes were not described, then the report of Eberhardt et al. (2004) provides an inaccurate description of these effects and of the theoretical validity of the conclusions and implications. The impetus to support theoretical ideas is on the original authors, but because the findings in Eberhardt et al. (2004) appear to be biased in favor of the researchers’ expectations, they do not provide such support.

### Goff et al. (2008) “Not Yet Human: Implicit Knowledge, Historical Dehumanization, and Contemporary Consequences”

This article reported six studies that purported to show an implicit association between Black Americans and apes. **Table 2** summarizes the statistical measures for each study, the key hypotheses that were tested in the article, and the estimated probability of success for those hypothesis tests.

**Table 2:** Statistical properties, hypotheses, and estimated probability of success for the tests in the six studies from Goff et al. (2008).

	Statistics	Supporting hypotheses	Probability of success
Study 1	*n_1_* = 41, *n_2_* = 40, *n_3_* = 40 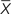 _1_ = 20.16, 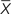 _2_ = 22.75, 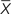 _3_ = 26.23 *s* = 5.05	μ_1_ ≠ μ_2_ μ_3_ ≠ μ_2_ Joint	0.630 0.861 **0.507**
Study 2	*n_*1A*_* = 14, *n_*2A*_* = 15, *n_*1B*_* = 14, *n_*2B*_* = 15 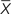 _*1A*_ = 1080, 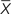 _*1B*_ = 2503 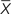 _*2A*_ = 3412, 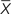 _*2B*_ = 1010 *s* = 2391	Interaction μ_1B_ ≠ μ_2B_ μ_1A_ ≠ μ_2A_ μ_1A_ ≠ μ_1B_ μ_2A_ ≠ μ_2B_ Joint	≈1.00 0.993 0.916 0.975 0.968 **0.879**
Study 3	*n_*1A*_* = 12, *n_*2A*_* = 12, *n_*1B*_* = 12, *n_*2B*_* = 13 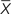 _*1A*_ = 625, 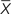 _*1B*_ = 874 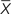 _*2A*_ = 814, 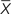 _*2B*_ = 801 *s* = 2999	Interaction μ_1B_ = μ_2B_ μ_1A_ ≠ μ_2A_ μ_1A_ ≠ μ_1B_ μ_2A_ = μ_2B_ Joint	0.684 0.948 0.905 0.762 0.882 **0.500**
Study 4	*n_1_* = 32, *n_2_* = 33 *F* = 30.46	μ_1_ ≠ μ_2_	**≈1.00**
Study 5	*n_*1A*_* = 29, *n_*2A*_* = 29, *n_*1B*_* = 29, *n_*2B*_* = 28 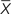 _*1A*_ = 3.88, 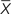 _*1B*_ = 2.86 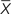 _*2A*_ = 2.90, 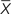 _2B_ = 3.13 *s* = 1.49	Interaction μ_1B_ = μ_2B_ μ_1A_ ≠ μ_2A_ μ_2A_ = μ_2B_ μ_1A_ ≠ μ_1B_ Joint	0.608 0.896 0.701 0.910 0.736 **0.381**
Study 6	*n_1_* = 15, *n_2_* = 138 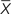 _1_ = 2.2, *s_1_* = 2.34 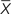 _2_ = 8.53, *s_2_* = 12.35	μ_1_ ≠ μ_2_	**0.565**
*P_TES_*			0.048

Study 1 measured participant’s sensitivity to apes or other animals in short degraded movies. Participants varied in priming condition (Black faces, White faces, none) and race of participant (White, non-White). Comparisons were also made within-subjects for type of animal (ape or non-ape). The primary hypotheses were for the ape trials: (1) participants were significantly more sensitive to ape movies when primed by Black faces than by no prime, and (2) participants were significantly less sensitive to ape movies when primed by White faces than by no prime. Goff et al. (2008) did include other (successful) hypothesis tests, but the within-subjects structure of the experiment prohibits calculating success probabilities for all of the tests because standard reporting practices do not provide enough information (e.g., correlations between within-subject measures were not reported). The estimated probability of a random sample of participants satisfying both tests is just slightly above one half. Since there were additional successful tests, this value likely overestimates the probability of replication experiments being as successful as the original study.

Study 2 reported evidence that participants primed with apes would have an attentional bias toward Blacks. The experiment varied prime type (apes or none) and a dot-probe positioned on a type of face (Black or White) in a between subjects design. Support for the theoretical ideas was provided by the following hypothesis tests: (1) a significant interaction between prime type and face type, (2) a significant effect of face type for the no prime condition, (3) a significant effect (in the opposite direction) of face type for the ape prime condition, (4) a significant effect of prime type in the Black face condition, and (5) a significant effect (in the opposite direction) of prime type in the White face condition. **Table 2** indicates that the probability of all these hypothesis tests being successful in a single sample is around 0.879. Following the analysis in Goff et al. (2008), the mean values in **Table 2** are the measured latencies, but the analysis was based on the reciprocal of the latencies. The pooled standard deviation was computed from the reciprocal means and reported *F*-values. **Table 2** shows that the estimated success probabilities are quite high for each hypothesis test. The joint success probability is also only somewhat smaller.

Study 3 was similar to study 2, but used an Asian face rather than a White face. The theoretical ideas were supported by five hypothesis tests: (1) a significant interaction between prime type and face type, (2) a non-significant effect of face type for the no prime condition, (3) a significant effect of face type for the ape prime condition, (4) a significant effect of prime type in the Black face condition, and (5) a non-significant effect of prime type in the Asian face condition. **Table 2** shows that although each test has a success probability comfortably above one-half, the probability of all five tests being successful in a single sample is only one half.

In Study 4 participants completed an Implicit Association Test that indicated faster categorization in a Black-ape condition than in a Black-big cat condition. The key test was for the relationship to hold even when co-varying for effects of a personalized IAT. The probability of success for this result can be estimated by converting the reported *F*-value to a standardized effect size.

In Study 5 participants judged violence justification in a video showing police beating a target suspect that was suggested to be either White or Black. Different groups of participants were also primed for apes or big cats. The theoretical ideas were supported by five hypothesis tests: (1) a significant interaction between prime type and target race, (2) a non-significant effect of prime type when the target was White, (3) a significant effect of prime type when the target was Black, (4) a non-significant effect of target race when the prime was big cats, and (5) a significant effect of target race when the prime was apes. The probability of a sample producing a successful outcome for all of these effects is 0.381.

Study 6 examined news articles discussing death-penalty eligible cases and reported a significant difference between the number of ape-related words in articles for Black defendants and White defendants. This test has a success probability just a bit higher than one half. The analysis included some secondary hypothesis tests on subsets of the data, but it is not possible to estimate the joint success probabilities for the full set of tests because the subset sample sizes are not fully described.

Even though two of the studies in Goff et al. (2008) have quite high success probabilities, the other four studies have low to modest success probabilities. The theoretical ideas in Goff et al. (2008) depend on the success of all the presented results, and the probability that six studies like these would all be uniformly successful is the product of the joint success probabilities, which is 0.048. This value is an estimate of the probability that a set of replication experiments with the same sample sizes would be as successful as the studies in Goff et al. (2008). The value is low enough that scientists should be skeptical about the validity of the experimental results or the theoretical ideas presented in Goff et al. (2008).

### Williams and Eberhardt (2008) “Biological Conceptions of Race and the Motivation to Cross Racial Boundaries”

This article reported five studies that purported to show that perceiving and interacting with racial outgroups was influenced by whether views of race were socially or biologically based. **Table 3** summarizes the statistical measures for each study, the key hypotheses that were tested in the article, and the estimated probability of success for those hypothesis tests.

**Table 3:** Statistical properties, hypotheses, and estimated probability of success for the tests in the five studies from Williams and Eberhardt (2008).

	Statistics	Supporting hypotheses	Probability of success
Study 1	*n* = 129, *r* = 0.19	ρ ≠ 0	**0.579**
Study 2	*n_1_* = 40, *n_2_* = 40 *F* = 3.38	μ_1_ ≠ μ_2_*	**0.496**
Study 3	*n* = 507, *r* = -0.26	ρ ≠ 0	**≈1.00**
Study 4	*n_*1A*_* = 43, *n_2A_* = 57, *n_3A_* = 29 *n_*1B*_* = 41, *n_*2B*_* = 55, *n_*3B*_* = 28 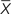 _*1A*_ = 2.87, 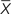 _*2A*_ = 3.37, 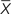 _*3A*_ = 3.55 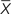 _*1B*_ = 3.21, 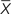 _*2B*_ = 3.38, 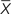 _*3B*_ = 3.30 *s* = 1.21	μ_iA_ ≠ μ_jA_ μ_1A_ ≠ μ_2A_ μ_1A_ ≠ μ_3A_ μ_iB_ = μ_jB_ Joint	0.614 0.636 0.526 0.915 **0.367**
Study 5	*n_1_* = 75, *n_2_* = 76 *p_1_* = 0.40, *p_2_* = 0.59	P_1_ ≠ P_2_	**0.661**
*P_TES_*			0.070

Study 1 reported a significant correlation between a race conception scale (RCS) and average race disparity scores even when controlling for participant scores on a Modern Racism Scale. The RCS was designed to measure people’s conceptions of race as being biologically based. This significant correlation was used to support the theoretical ideas. However, an experiment like this one is only estimated to be successful around 60% of the time. Several other successful significance tests were also presented as support for the theoretical ideas, but it is not possible to estimate their joint success probability.

Study 2 primed participants to think of race as having a biological or social basis and then measured emotional engagement. The observed difference in mood was reported as support for the theoretical ideas. Williams and Eberhardt (2008) used a non-standard significance criterion (*p* < 0.07), so the same criterion was used to estimate the success probability for an experiment of this type. The power of an experiment like this can be estimated from the standardized effect size, which can be computed from the provided *F*-value.

Study 3 reported a significant negative correlation between RCS scores and self-reported motivation for contact with diverse others. The estimated success probability for an experiment like this is quite high.

Study 4 assigned participants to a three by two design (three prime types: biological, social, control; two target race: same, other) and measured friendship motivation scores. Support for the theoretical ideas was based on several hypothesis tests: (1) a significant effect of priming condition for participants in the other race condition, (2) lower friendship motivation scores for other race condition participants in the biological condition than in the social condition, (3) lower friendship motivation scores for other race participants in the biological condition than in the control condition, (4) non-significant findings for priming among participants in the same race condition. The estimated probability of all tests producing a successful outcome is around one-third.

Study 5 primed participants for either a biological or social conception of race. Participants were then told that they were assigned to work with a Black partner on a task and reported their willingness to sign up for future sessions with the same partner. The proportion of participants willing to sign up for future sessions was significantly lower for those primed with a biological conception than for those primed for a social conception, but the estimated probability of rejecting the null hypothesis is around two-thirds.

Each but one of the studies in Williams and Eberhardt (2008) has a low to modest joint success probability. The probability that five studies like these would all be uniformly successful is the product of the joint success probabilities, which is 0.070; and the low value suggests that the reported degree of success is unlikely to be replicated by future studies with the same sample sizes and design. Indeed, the probability is low enough that scientists should doubt the validity of the experimental results and the theoretical ideas presented in Williams and Eberhardt (2008).

## Discussion

Racial bias and racial discrimination are important and complex topics. Society needs scientific investigations that provide good evidence about the basis and properties of racial bias, but given the apparent excess success in Eberhardt et al. (2004), Goff et al. (2008), and Williams and Eberhardt (2008), scientists should be skeptical about the experimental findings and theoretical claims presented in those articles. If the effects were similar to what was reported and the studies were run, analyzed, and reported unbiasedly, then the observed degree of success in each article would be quite rare. This rarity suggests that the findings were not run, analyzed, or reported unbiasedly; and thus scientists should have some skepticism about the merit of the proposed theoretical ideas.

It is important to recognize that the choices in experimental design, analysis, theorizing, and reporting for these articles may reflect standard methods in experimental psychology. For example, Francis (2014a) performed a TES analysis for all recent articles with four or more experiments that were published in the prestigious journal *Psychological Science*. The analyses found that 36 out of 44 articles (82%) failed the TES. A corresponding analysis of psychology-related articles in the journal *Science* (Francis et al., 2014) found a similar rate of excess success (83%).

It is regrettable that the efforts of Eberhardt and colleagues, however well intentioned and however similar to standard procedures, have resulted in experimental findings and theories that do not improve our understanding of such an important topic. Since these articles do not provide good scientific support for the theoretical ideas, it seems premature to apply these ideas to policy decisions or to interventions that might reduce the presence or impact of racial bias. Such applications cannot be justified on scientific grounds.
